# Supplementary material for: RNA-Seq Analysis Identifies Transcription Factors Involved in Anthocyanin Biosynthesis of ‘Red Zaosu’ Pear Peel and Functional Study of PpPIF8
Source: Int J Mol Sci. 2022 Apr 27;23(9):4798. doi: 10.3390/ijms23094798 (PMC9099880; doi:10.3390/ijms23094798)
Supplement: Supplementary file 1 [file ijms-23-04798-s001.zip › ijms-1687962-supplementary figures.pdf]

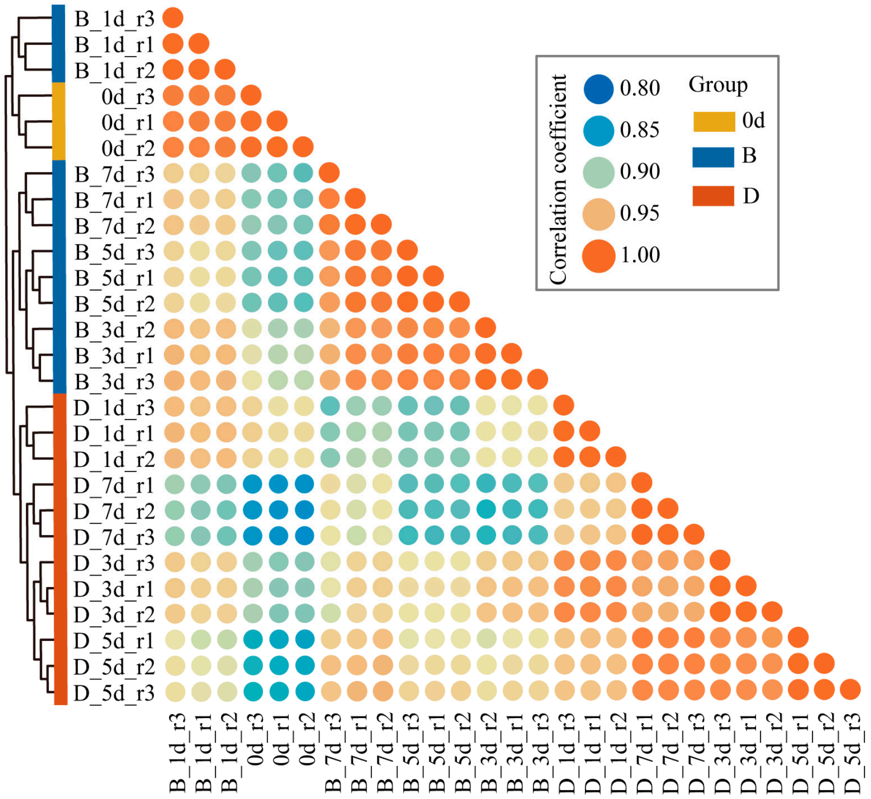

Figure S1.Spearman correlation and clustering analysis of sequencing sample.

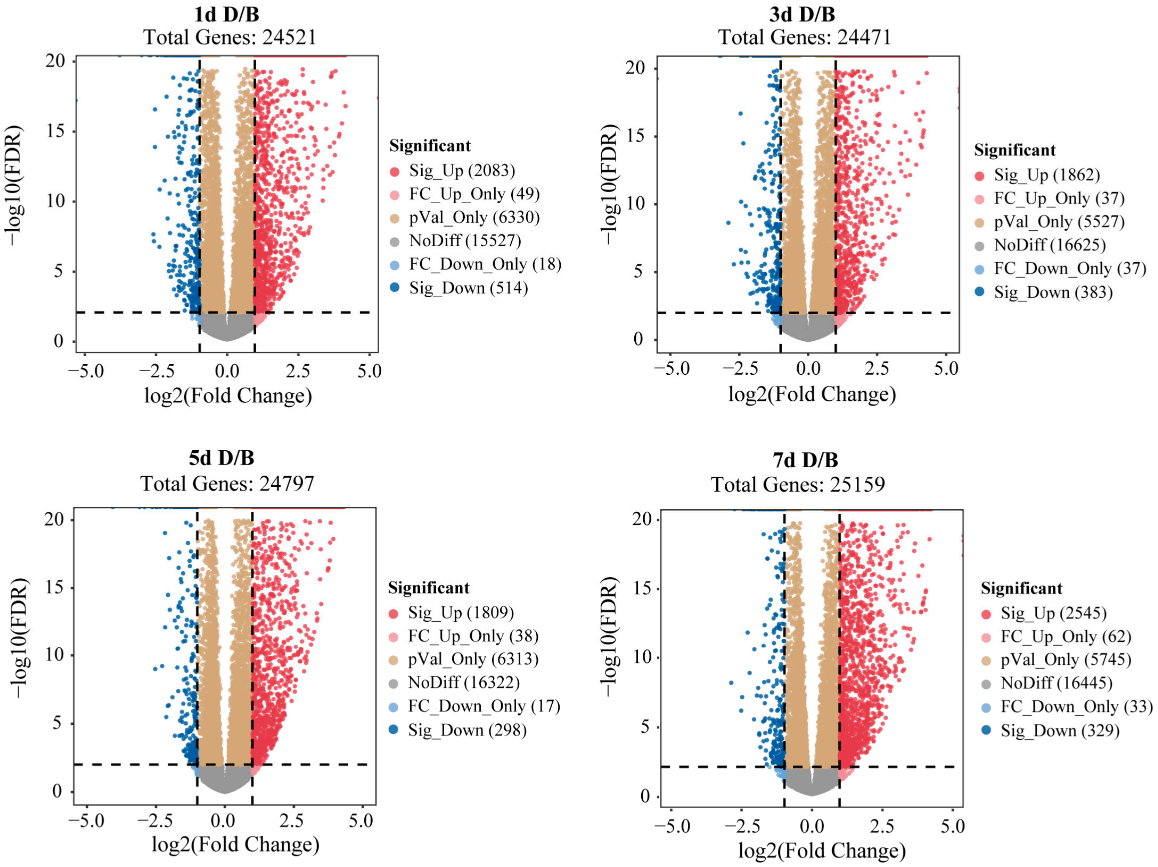

Figure S2.Volcano plots showing the differential gene expression between D and B group samples at different timepoint. Genes significantly upregulated and downregulated in D group samples are shown in red and blue, respectively.

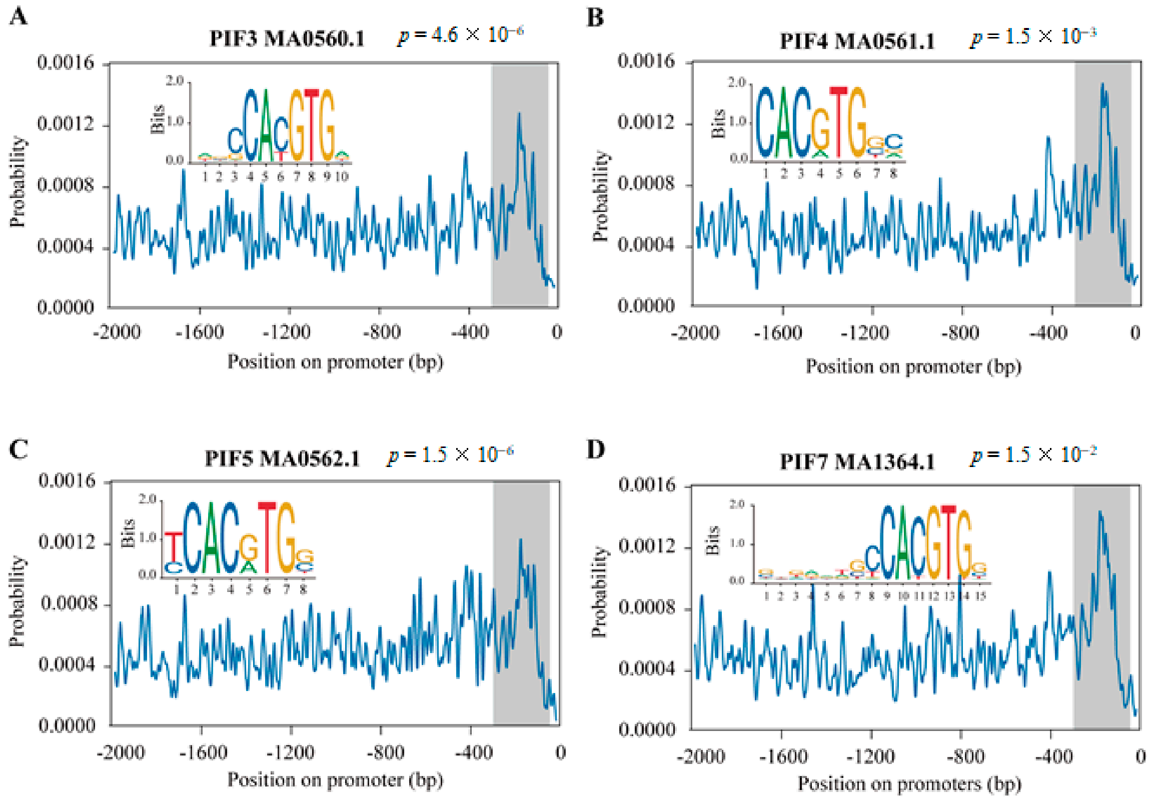

Figure S3.The distribution preference of the PIF3, PIF4, PIF5, and PIF7 binding site in the promoter region of genes in the "Blue" and "Turquoise" modules.
