# Supplementary material for: RNA-Seq Analysis Identifies Transcription Factors Involved in Anthocyanin Biosynthesis of ‘Red Zaosu’ Pear Peel and Functional Study of PpPIF8
Source: Int J Mol Sci. 2022 Apr 27;23(9):4798. doi: 10.3390/ijms23094798 (PMC9099880; doi:10.3390/ijms23094798)
Supplement: Supplementary file 1 [file ijms-23-04798-s001.zip › Table S1.Primers used in this study.pdf]

Table S1 Primers used in this study.

**Primers used for DNA constructs**		
Primer Name	Forward (5'-3')	Reverse (5'-3')
PpPIF8-pTOPO	caccATGAGCCAGCGCGTTCCTAGC	GCCCTTAGAGCTAGATGCTGGAG
PpPIF8-GFP	agaacacgggggactctagaATGAGCCAGCGCGTTCCTAGC	cccttgctcaccatggatccGCCCTTAGAGCTAGATGCTGGAG
**Primes used for *PpCHS* Genome walker**		
Primer Name	5'-3'	
ProPpCHS-GSP1	TTGATCCACACAGTTGGGAGGAGTTG	
ProPpCHS-GSP2	TGAGCCTTGCGAACTTCCTCGACAGTC	
**Primers used for RT-qPCR**		
Gene Name	Forward (5'-3')	Reverse (5'-3')
PpPIF8	ATGCCAGGGGTGATTTCTGG	GAAGGGTGTCATCGTCGTGT
PpPAL	TCCATGGACAACACTCGCTT	AGAAACTGCAGCTCGGAACA
PpC4H	AACTTCGAGCTTCTGCCTCC	CCCCAAGCATCAATCTACGC
Pp4CL	GATCCGGAGTCGACAAGGAC	GGGCCACTTGAAACCCTTTG
PpCHS	TACCGTCGAGGAAGTTCGCA	TCGGGATATGTGGCTTGATCC
PpCHI	GCGGTTACATGCAATCCCAG	CAGTGCCCATCTTACTGGCT
PpF3H	CAGAAGCCATGGGGTTGGAT	CCAAGAGTTAGGTCGGGCTG
PpF3'H	CTCCCTAGAGTGGCTGGACT	TCCTTGAGCGACAGCAATGT
PpF3'5'H	ATACCAGTTCCGGGTGGAGT	GAAGTGCTGGCGTTTTGAGG
PpFLS	GCCGGTGGTGATAACTTGGA	TGAACATCGTTGGGGACGAG
PpDFR	GGCATCTGTTCGCGACATTC	AAAGCCTCGGACTTGTGGAG
PpANS	CCCACCACGAACTTTTGCTG	ATTTAGCACAACCCGCCTCA
PpUFGT	CTTCAAATGGGTCGGCTCCT	ATGGAGCGTCTTGCTTGTCA
