# Supplementary material for: RNA-Seq Analysis Identifies Transcription Factors Involved in Anthocyanin Biosynthesis of ‘Red Zaosu’ Pear Peel and Functional Study of PpPIF8
Source: Int J Mol Sci. 2022 Apr 27;23(9):4798. doi: 10.3390/ijms23094798 (PMC9099880; doi:10.3390/ijms23094798)
Supplement: Supplementary file 1 [file ijms-23-04798-s001.zip › Table S2.Sequencing data Statistics and Alignment results of each sample.pdf]

Table S2. Sequencing data Statistics

Samples	Clean reads	Clean bases	GC Content	%≥Q30
0d_r1	24,045,281	7,182,026,092	46.89%	94.08%
0d_r2	22,431,782	6,698,837,974	47.19%	94.26%
0d_r3	23,661,897	7,076,303,010	46.84%	93.78%
D_1d_r1	22,169,449	6,620,661,824	47.00%	94.18%
D_1d-r2	20,372,847	6,085,888,904	47.11%	94.31%
D_1d-r3	22,776,951	6,807,991,040	47.31%	94.37%
D_3d-r1	22,558,110	6,752,424,186	46.81%	93.73%
D_3d-r2	22,158,902	6,630,899,756	46.94%	93.88%
D_3d-r3	23,306,938	6,977,305,576	46.96%	94.13%
D_5d-r1	25,610,115	7,661,973,392	46.91%	93.42%
D_5d-r2	22,006,975	6,584,487,138	46.90%	93.88%
D_5d-r3	24,119,410	7,213,856,598	47.03%	93.87%
D_7d-r1	23,391,777	6,998,400,718	46.74%	93.62%
D_7d-r2	24,478,106	7,326,402,512	46.77%	93.35%
D_7d-r3	21,966,094	6,577,158,950	46.68%	93.36%
B_1d-r1	24,363,719	7,283,561,696	47.22%	94.51%
B_1d-r2	23,273,373	6,939,629,100	47.20%	94.53%
B_1d-r3	23,887,617	7,131,761,964	47.32%	94.26%
B_3d-r1	23,455,470	7,008,048,408	47.26%	94.03%
B_3d-r2	24,058,239	7,189,730,008	47.00%	94.01%
B_3d-r3	21,801,574	6,512,960,250	46.91%	93.90%
B_5d-r1	21,924,147	6,545,456,728	47.04%	94.18%
B_5d-r2	21,906,947	6,543,301,272	47.23%	94.28%
B_5d-r3	22,912,551	6,844,933,142	47.11%	93.87%
B_7d-r1	21,914,247	6,549,980,300	46.98%	93.76%
B_7d-r2	22,127,007	6,603,514,010	47.02%	94.25%
B_7d-r3	21,459,140	6,417,388,280	46.42%	92.97%

Alignment results of each sample

Samples	Total Reads	Mapped Reads	Uniq Mapped Reads	Multiple Map Reads
0d_r1	48,090,562	36,748,993 (76.42%)	68.84%	7.57%
0d_r2	44,863,564	34,362,610 (76.59%)	69.05%	7.55%
0d_r3	47,323,794	36,016,166 (76.11%)	68.49%	7.62%
D_1d_r1	44,338,898	33,811,671 (76.26%)	68.86%	7.39%
D_1d-r2	40,745,694	31,254,235 (76.71%)	69.27%	7.44%
D_1d-r3	45,553,902	34,968,887 (76.76%)	69.35%	7.41%
D_3d-r1	45,116,220	34,148,773 (75.69%)	68.46%	7.23%
D_3d-r2	44,317,804	33,582,095 (75.78%)	68.53%	7.25%
D_3d-r3	46,613,876	35,293,217 (75.71%)	68.51%	7.21%
D_5d-r1	51,220,230	38,591,963 (75.35%)	68.11%	7.24%
D_5d-r2	44,013,950	33,200,823 (75.43%)	68.14%	7.29%
D_5d-r3	48,238,820	36,543,599 (75.76%)	68.39%	7.36%

D_7d-r1	46,783,554	35,426,691 (75.72%)	68.42%	7.30%
D_7d-r2	48,956,212	36,669,962 (74.90%)	67.63%	7.27%
D_7d-r3	43,932,188	33,259,502 (75.71%)	68.59%	7.12%
B_1d-r1	48,727,438	37,388,407 (76.73%)	69.17%	7.56%
B_1d-r2	46,546,746	35,694,159 (76.68%)	69.06%	7.62%
B_1d-r3	47,775,234	36,410,225 (76.21%)	68.64%	7.57%
B_3d-r1	46,910,940	35,865,881 (76.46%)	68.72%	7.73%
B_3d-r2	48,116,478	36,714,197 (76.30%)	68.68%	7.62%
B_3d-r3	43,603,148	33,181,291 (76.10%)	68.50%	7.59%
B_5d-r1	43,848,294	33,502,809 (76.41%)	68.77%	7.64%
B_5d-r2	43,813,894	33,520,211 (76.51%)	68.80%	7.70%
B_5d-r3	45,825,102	34,954,771 (76.28%)	68.63%	7.65%
B_7d-r1	43,828,494	33,388,593 (76.18%)	68.61%	7.57%
B_7d-r2	44,254,014	33,782,630 (76.34%)	68.71%	7.63%
B_7d-r3	42,918,280	32,532,412 (75.80%)	68.37%	7.43%
